# Personalized Target Heart Rate for Patients with Heart Failure and Reduced Ejection Fraction

**DOI:** 10.3390/jpm12010050

**Published:** 2022-01-05

**Authors:** Yusuke Yumita, Yuji Nagatomo, Makoto Takei, Mike Saji, Ayumi Goda, Takashi Kohno, Shintaro Nakano, Yosuke Nishihata, Yukinori Ikegami, Yasuyuki Shiraishi, Shun Kohsaka, Takeshi Adachi, Tsutomu Yoshikawa

**Affiliations:** 1 Department of Cardiology, National Defense Medical College, Tokorozawa 359-8513, Japan; doc33073@ndmc.ac.jp (Y.Y.); con467@ndmc.ac.jp (Y.I.); tadachibu@gmail.com (T.A.); 2 Department of Cardiology, Sakakibara Heart Institute, Tokyo 183-0003, Japan; mikesaji8@gmail.com (M.S.); tyoshi@shi.heart.or.jp (T.Y.); 3 Department of Cardiology, Saiseikai Central Hospital, Tokyo 108-0073, Japan; makoto_tk@hotmail.com; 4 Department of Cardiovascular Medicine, Faculty of Medicine, Kyorin University, Tokyo 181-8611, Japan; ayumix34@yahoo.co.jp (A.G.); kohno-ta@ks.kyorin-u.ac.jp (T.K.); 5 Department of Cardiology, International Medical Center, Saitama Medical University, Hidaka 350-1298, Japan; snakano@saitama-med.ac.jp; 6 Department of Cardiology, St. Luke’s International Hospital, Tokyo 104-8560, Japan; hatasuke@luke.ac.jp; 7 Department of Cardiology, National Hospital Organization Tokyo Medical Center, Tokyo 152-8902, Japan; 8 Department of Cardiology, Keio University School of Medicine, Tokyo 160-8582, Japan; yasshiraishi@keio.jp (Y.S.); sk@keio.jp (S.K.)

**Keywords:** heart rate, mitral inflow, heart failure with reduced ejection fraction

## Abstract

The optimal heart rate (HR) in patients with heart failure with reduced ejection fraction (HFrEF) has been ill-defined. Recently, a formula was proposed for estimating the target heart rate (THR), which eliminates the overlap between the E and A wave (E-A overlap). We aim to validate its prognostic significance in the multicenter WET-HF registry. This study used data from 647 patients with HFrEF hospitalized for acute decompensated HF (ADHF). The patients were divided into the 2 groups by THR. The primary endpoint was defined as the composite of all-cause death and ADHF readmission. The THR successfully discriminated the incidence of the primary endpoint, whereas no significant difference was observed in the primary endpoint when dividing the patients by uniform cutoff 70 bpm. HR at discharge ≤ THR was inversely associated with the primary endpoint. Restricted cubic spline analysis demonstrated the difference between HR at discharge, and THR (ΔHR) from −10 to ±0 was associated with a lower risk of primary endpoint and ΔHR from ±0 to +15 was associated with a higher risk. THR discriminated long-term outcomes in patients with HFrEF more efficiently than the uniform cutoff, suggesting that it may aid in tailored HR reduction strategies.

## 1. Introduction

Heart failure (HF) is a major societal problem that has been increasing in prevalence worldwide. Although novel agents and devices have been developed to manage HF, their clinical outcome has not sufficiently improved in Japan for over a decade [1].

Heart rate (HR) is an important hallmark of long-term clinical outcomes in patients with HF and reduced ejection fraction (HFrEF). In HFrEF, a lower HR was associated with lower mortality [2]. HR reduction after β-blocker introduction was associated with more favorable outcomes such as lower mortality [3], which was evident especially in HFrEF patients with sinus rhythm (SR) [4,5]. In systolic heart failure treatment in the If inhibitor ivabradine (SHIFT) trial, which examined the safety and efficacy of the sinus node inhibitor ivabradine in patients with HFrEF and SR, ivabradine reduced the primary composite endpoint of cardiovascular death or admission for acute decompensated heart failure (ADHF) compared to the placebo group [6]. In this study, ivabradine administration showed a significant reduction in HR [6], and a lower HR achieved at 28 days was associated with a lower incidence of the primary endpoint [7]. However, the optimal HR for a given patient with HFrEF has not been investigated and remains unclear.

A higher HR is associated with a shorter left ventricular (LV) diastolic time duration, which results in an overlap between the E wave and A wave (E-A overlap) in mitral inflow [8]. A larger E-A overlap was associated with higher LA pressure at diastole [9]. These findings suggest that E-A overlap due to a higher HR might cause limited diastolic mitral inflow, which can lead to exaggerated hemodynamics and pulmonary congestion.

Recently, given the results of multiple linear regression analyses in HFrEF patients, the formula estimating HR (target HR [THR]), which eliminates E-A overlap, was proposed (THR [bpm] = 93 − 0.13 × deceleration time [ms], Central Illustration A) [10]. However, the prognostic significance of this formula in patients with HFrEF has not yet been validated. Hence, in the present study, we aimed to clinically evaluate the prognostic significance of THR in patients hospitalized for ADHF using data from the West Tokyo Heart Failure (WET-HF) registry.

## 2. Materials and Methods

### 2.1. Study Design

We analyzed data from 4000 patients with ADHF registered in the WET-HF registry from 2006 to 2017. The WET-HF registry is a multicenter prospective cohort registry that enrolled all patients hospitalized for ADHF according to the Framingham criteria [11]. Patients with acute coronary syndrome or isolated right-sided HF were excluded from the study. The clinical diagnosis of ADHF was made by individual cardiologists at each institution. The eight study centers were located in Tokyo, Japan, and included four university hospitals (Keio University, Kyorin University, Saitama Medical University, and National Defense Medical College) and four tertiary referral hospitals (Sakakibara Heart Institute, St. Luke’s International Hospital, Saiseikai Central Hospital, and National Hospital Organization Tokyo Medical Center).

Baseline data and outcomes for the WET-HF registry were collected by dedicated clinical research coordinators from medical records and interviews with treating physicians to obtain a robust assessment of the care and patient outcomes. Data were entered into an electronic data-capturing system with a robust data query engine and system validations for data quality. Outliers in continuous variables or unexpected values in the categorical variables were selected based on established criteria, and the originating institution was notified to verify the values. The quality of reporting was also verified by the principal investigators (Y.S. and S.K.) at least once a year, and periodic queries were conducted to ensure quality. Exclusive on-site auditing by the investigators (Y.S. and S.K.) ensured proper registration of each patient. Before the launch of the WET-HF registry, information regarding the objective of the present study, its social significance, and an abstract were provided for clinical trial registration with the University Hospital Medical Information Network (UMIN000001171). The study protocol was approved by the institutional review boards at each site, and the study was conducted in accordance with the Declaration of Helsinki. Ethical approval for the study was granted by the institutional review boards of Keio University Hospital (no. 20090176) and all participating institutions. Written and/or oral informed consent was obtained from each subject prior to the registration.

**Figure 1:**
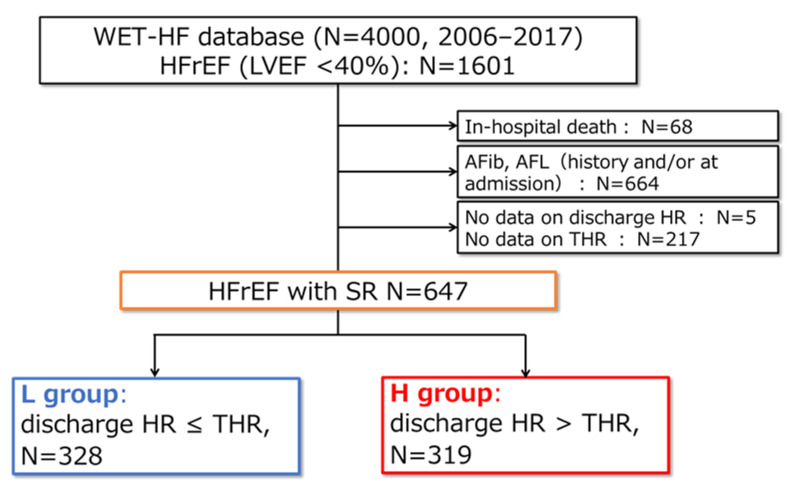
Flowchart of the study. The L group was defined as the patients whose discharge HR was less than or equal to the THR calculated by the following formula (THR [bpm] = 93 − 0.13 × Dct [ms]), and H group was defined as those whose discharge HR is higher than the THR. WET-HF: West Tokyo Heart Failure, HFrEF: heart failure with reduced ejection fraction, AFib: atrial fibrillation, AFL: atrial flutter, discharge HR: heart rate at discharge, THR: target heart rate, SR: sinus rhythm.

Figure 1 shows the flowchart of the present study. Among the 4000 patients, data from 1601 patients with HFrEF, defined as LVEF < 40%, were extracted. Patients who died during hospitalization (N = 68) and those who had a history or evidence of atrial fibrillation/flutter at admission were excluded (N = 664). Patients with no data on HR at discharge (discharge HR, N = 5) and those with no data on THR (N = 217) were also excluded. As a result, 647 patients with HFrEF and SR were included in the final analysis.

For each patient, THR was calculated using the following formula based on the deceleration time (Dct) value measured in the compensated HF phase during the index admission.

THR [bpm] = 93 − 0.13 × Dct [ms].

The patients were then divided into two groups: L group (discharge HR ≤ THR, N = 328) and H group (discharge HR > THR, N = 319) by THR.

### 2.2. Endpoint

A follow-up survey using medical charts or telephone interviews was performed, and patients who were lost to follow-up were censored at the date of last contact. Regarding HF readmission, treating physicians at each participating hospital made decisions according to the usual standard of care. The date of index hospitalization discharge, ADHF rehospitalization, mortality, and mode of death were collected and confirmed by site investigators and dedicated clinical research coordinators. The primary endpoint was defined as a composite of all-cause mortality and readmission for ADHF.

### 2.3. Statistical Analysis

Continuous variables are expressed as mean ± standard deviation for normally distributed data and as median (interquartile range) for data with non-normal distribution. Between-group differences were assessed using the unpaired *t*-test or Mann-Whitney U test for unpaired data. In contrast, the chi-squared test was used for the comparison of discrete variables. Kaplan–Meier survival curves were constructed for each group, and differences between groups were analyzed using the log-rank test. The beneficial effect of the HR-lowering strategy using ivabradine was demonstrated in patients with HR ≥70 bpm in the SHIFT study [6]. Therefore, THR and 70 bpm were employed as the cutoff values of discharge HR and compared in the present study. Cox proportional hazard models were used to assess the association with the primary endpoint. Age, sex, body mass index [BMI], New York Heart Association (NYHA) functional class, LVEF, and the independent variables that showed significant association with the primary endpoint by univariable analysis (estimated glomerular filtration rate [eGFR]; serum sodium [Na], hemoglobin [Hb], and albumin [Alb] levels; and β-blocker prescription) were employed in the multivariable analysis. The same analysis was conducted in the population stratified according to age, sex, BMI, ischemic etiology, diabetes mellitus, NYHA functional class, systolic blood pressure, LVEF, and eGFR. In addition, the relationship between discharge HR or “ΔHR” (calculated by subtracting THR from the discharge HR) was analyzed as a continuous variable, and the primary endpoint was modeled with a restricted cubic spline using four knots at the 5%, 35%, 65%, and 95% percentiles. The restricted cubic spline curves show the function relating discharge HR or ΔHR to the primary endpoint, where the discharge HR of 70 bpm or ΔHR of ±0 is set as the reference value (hazard ratio = 1). The aforementioned analyses were carried out using R version 4.1.0 (R Foundation for statistical computing, Vienna, Austria) with the “rms” package. Other statistical analyses were performed using JMP 14.2.0 (SAS Institute, Cary, NC, USA). Statistical significance was set at *p* < 0.05.

## 3. Results

The distribution of THR in the overall population, L group and H group, is shown in Supplementary Figure S1.

The baseline characteristics of the L and H groups are presented in Table 1 and Table 2. The patients in the L group showed a higher prevalence of males, higher BMI, more ICD implantation, and higher Hb and Alb levels. No significant differences in age, etiology of heart failure, previous heart failure hospitalization, or eGFR were observed between the two groups. Echocardiography revealed a larger left atrial diameter (LAD) and tricuspid regurgitation pressure gradient (TRPG) and smaller Dct in the L group. LVEF and E/e’ were similar in both groups. At discharge, HR was lower, and the THR calculated from the Dct was higher in the L group, although no significant differences were found in β-blocker prescription or dose.

### 3.1. Long-Term Outcomes in the L and H Groups

**Figure 2:**
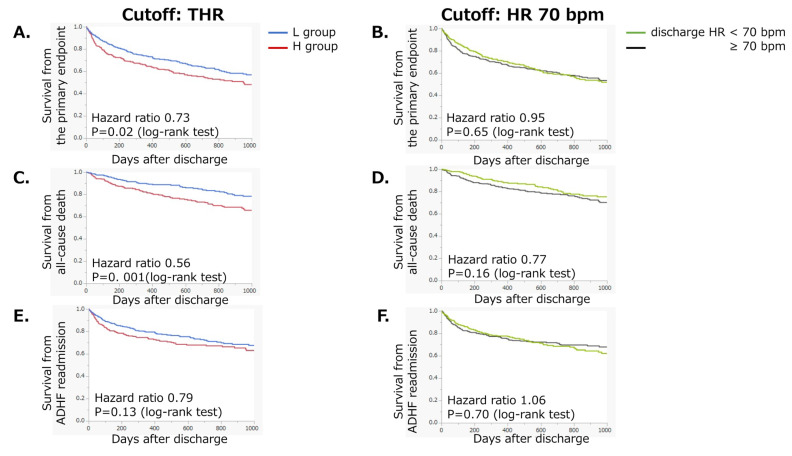
Kaplan-Meier curves for (**A**,**B**) the primary endpoint, (**C**,**D**) all-cause death, and (**E**,**F**) ADHF readmission dividing the study population according to discharge HR by a cutoff of THR or 70 bpm. The primary endpoint was defined as a composite of all-cause death and ADHF readmission. ADHF: acute decompensated heart failure, discharge HR: heart rate at discharge, THR: target heart rate.

A total of 125 deaths (L group, 50; H group, 75) and 165 ADHF readmissions (L group, 79; H group, 86) occurred during the first 1000 days after discharge. In the Kaplan-Meier curve, the L group showed a significantly lower rate of the primary endpoint (*p* = 0.018, log-rank test, Figure 2A), defined as the composite of all-cause death and readmission for ADHF. In contrast, no significant difference in the incidence of the primary endpoint divided by a uniform cutoff value of discharge HR of 70 bpm was found between the two groups (Figure 2B). All-cause death was also less common in the L group (*p* = 0.001, Figure 2C), whereas no significant difference was observed between the two groups when divided by a uniform cutoff value of discharge HR of 70 bpm (Figure 2D). No significant difference in cardiac death was found between the L and H groups (*p* = 0.17); however, there was a significant difference in non-cardiac death (L group, 11; H group, 27, *p* = 0.005). ADHF readmission rate did not significantly differ when dividing the study population by THR (Figure 2E) or 70 bpm (Figure 2F).

**Figure 3:**
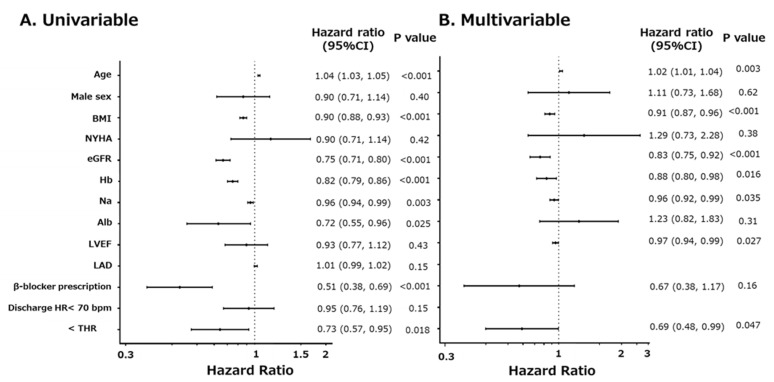
(**A**) Univariable and (**B**) multivariable Cox proportional hazard model analysis for the primary endpoint. BMI, body mass index; NYHA, New York Heart Association functional class; eGFR, estimated glomerular filtration rate; Hb, hemoglobin level, Na, serum sodium level, Alb, serum albumin level; LVEF, left ventricular ejection fraction; LAD, left atrial diameter; discharge HR, HR at discharge; THR, target HR.

Cox proportional hazards model analysis was performed to determine the independent association of the variables with the primary endpoint (Figure 3). Univariable analysis revealed that age, low BMI, low eGFR, low hemoglobin (Hb) level, low serum Na level, low serum Alb level, no β-blocker prescription at discharge, and discharge HR ≤ THR showed significant association with the primary endpoint (Figure 3A). Conversely, discharge HR < 70 bpm was not significantly associated with the primary endpoint (Figure 3A). Multivariable analysis revealed that the association of discharge HR ≤ THR with the primary endpoint remained significant after adjustment for covariates (Figure 3B).

### 3.2. Restricted Cubic Spline Curves for the Function Relating Discharge HR or THR and the Endpoints

**Figure 4:**
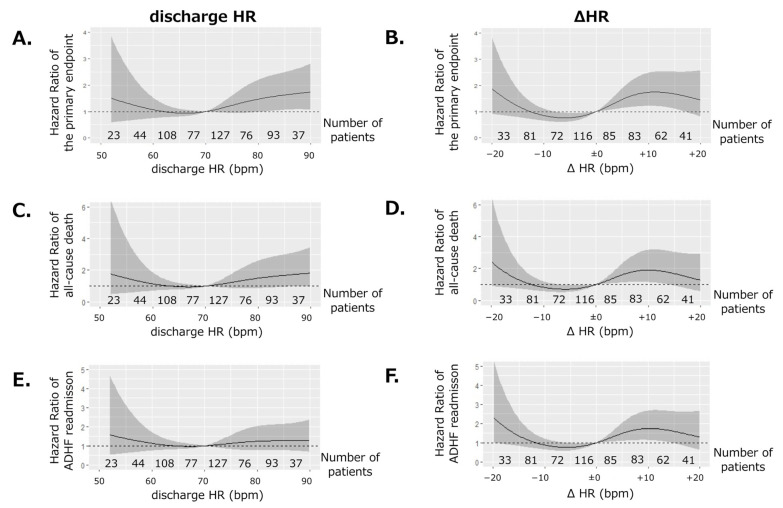
Restricted cubic spline analysis depicting the association of discharge HR or ΔHR with a hazard ratio of (**A**,**B**) the primary endpoint, (**C**,**D**) all-cause death, and (**E**,**F**) ADHF readmission. ΔHR was defined as the difference between discharge HR and THR. Hazard ratio was adjusted for the covariates shown in Figure 3B (age; sex; BMI; NHYA; eGFR; Na, Hb, and Alb levels; LVEF; and β-blocker prescription). HR: heart rate at discharge, THR: target heart rate, BMI: body mass index, NYHA: New York Heart Association functional class, eGFR: estimated glomerular filtration rate, Hb: hemoglobin level, Na: serum sodium level, Alb: serum albumin level, LVEF: left ventricular ejection fraction.

To further clarify the difference in risk stratification ability between discharge HR and THR, a restricted cubic spline analysis of discharge HR or ΔHR and the primary endpoint was performed. Figure 4A shows the association between discharge HR and the hazard ratio of the primary endpoint. A significant increase or decrease in hazard ratio was scarcely observed across the entire spectrum of discharge HR when using HR of 70 bpm as a reference. Conversely, when using ΔHR ± 0 as a reference, a significant association of ΔHR from −10 to ±0 with a lower hazard ratio and that of ΔHR from ±0 to +15 with a higher hazard ratio was observed (Figure 4B). Similarly, a significant increase or decrease in the risk of all-cause death (Figure 4C) or ADHF readmission (Figure 4E) was scarcely observed across the entire spectrum of discharge HR. In contrast, a significant association of ΔHR from −10 to ±0 with lower risk and ΔHR from ±0 to +15 with a higher risk of all-cause death (Figure 4D) or ADHF readmission (Figure 4F) was observed. However, the association of ΔHR with ADHF readmission seemed to be relatively weak (Figure 4F).

### 3.3. Stratified Analysis

Supplementary Figure S2 shows the hazard ratios of the L/H group for the primary endpoint adjusted for the covariates (age; sex; BMI; NYHA functional class; eGFR; Hb, Na, and Alb levels; LVEF and β blocker prescription) in the subgroups. Discharge HR ≤ THR was associated with a lower rate of the primary endpoint in the following subgroups: patients aged ≥ 70 years (HR 0.630, 95% CI 0.405–0.981, *p* = 0.041), male (HR 0.618, 95% CI 0.386–0.989), BMI ≥ 25 (HR 0.147, 95% CI 0.053–0.408, *p* < 0.001), ischemic etiology (HR 0.568, 95% CI 0.337–0.958, *p* = 0.034), NYHA class III/IV (HR 0.676, 95% CI 0.458–0.998, *p* = 0.049), and SBP ≥ 140 (HR 0.469, 95% CI 0.242–0.910, *p* = 0.025; Supplementary Figure S2). Notably, BMI significantly modified the influence of discharge HR ≤ THR on the incidence of the primary endpoint (*p* for interaction = 0.030).

## 4. Discussion

This study demonstrated the following main findings: (1) Achievement of THR was associated with a lower incidence of the primary endpoint, defined as the composite of all-cause death and ADHF rehospitalization. (2) Cox proportional hazard model analysis indicated that the achievement of THR was independently associated with the primary endpoint. (3) The restricted cubic spline analysis demonstrated that ΔHR −10 to 0 had the lowest incidence of the primary endpoint, all-cause death, and ADHF readmission.

Based on these findings, we concluded that THR successfully discriminated subsequent long-term clinical outcomes in patients with HFrEF and SR.

### 4.1. Association of HR with E-A Overlap and Hemodynamics

The previous study demonstrated that HR reduction through ivabradine administration did not affect cardiac output [12]. This is explained by the compensatory mechanism of LV volume expansion, which increases stroke volume leading to preserved cardiac output [12]. In addition, HR increase by atrial pacing did not affect cardiac output in humans [13]. While increased HR by atrial pacing did not affect either the E wave or A wave duration, it decreased the duration of diastasis [14]. Thus, an increase in HR eventually causes an E-A overlap. A larger E-A overlap correlated well with higher pulmonary capillary wedge pressure [15]. Taken together, while increased HR could affect cardiac output minimally, it could cause E-A overlap, as demonstrated through pulsed-wave Doppler, which might result in limited mitral inflow. These sequences might aggravate the efficiency of cardiac work, resulting in worsening of reverse cardiac remodeling, leading to subsequent clinical adverse events. Indeed, in a recent study, a larger E-A overlap was shown to be associated with subsequent ADHF hospitalization [15]. The difference between discharge HR and THR was shown to be associated with LVEF improvement after one year [16].

### 4.2. Clinical Significance of THR in Patients with HFrEF

Our data suggest that achieving THR at discharge was associated with a lower incidence of the primary endpoint (Figure 2 and Figure 3, and Graphic Abstract), whereas achieving a discharge HR of 70 bpm was not. Based on these findings, the target HR might be determined on a per-patient basis. The THR was simply calculated using the formula assigning Dct. A higher THR corresponds to a decreased Dct, indicating impaired LV diastolic function [17]. In such cases, a relatively higher HR might be acceptable compared to patients with preserved LV diastolic function.

In contrast, the difference between discharge HR and THR of −10 to ±0 was associated with the lowest incidence of the primary endpoint (Figure 4 and Graphic Abstract). It is well known that too slow HR due to bradyarrhythmia, such as sick sinus syndrome or advanced AV block per se, can cause ADHF [18]. Therefore, it is conceivable that HR much lower than THR can exacerbate hemodynamics, which can lead to deleterious consequences. If this is the case, the “the lower, the better” theory cannot be applicable for HR reduction strategies in the management of patients with HFrEF with SR. To elucidate this issue, ideally, a randomized study that compares the strategies aimed at a certain HR (e.g., 50 bpm, 70 bpm) and THR would be needed.

The association between achieving THR and the incidence of ADHF readmission was not statistically significant (Figure 2E). This might be due to less optimized medication, including HR-lowering agents, during the index hospitalization since this registry enrolled patients hospitalized for ADHF. However, in the restricted cubic spline analysis, the association of ΔHR with the hazard ratio for ADHF readmission showed a trend similar to that with the primary endpoint or all-cause mortality (Figure 4B,D,F). These findings support the prognostic significance of THR for all-cause mortality and readmission in patients with HFrEF.

### 4.3. Association of Achieving THR and BMI in Patients with HFrEF

In the stratified analysis, discharge HR ≤ THR in the subgroup with BMI ≥ 25 kg/m^2^ was significantly correlated with a lower incidence of the primary endpoint (Supplementary Figure S2). The subgroup with BMI ≥ 25 kg/m^2^ was associated with younger age, higher prevalence in men and of DCM, a lower proportion of ADHF admission history, lower B-type natriuretic peptide (BNP)/N-terminal proBNP, and higher Hb level and eGFR (data not shown). However, the β-blocker dose per body weight at discharge was similar between the patients with BMI < 25 kg/m^2^ and ≥25 kg/m^2^, a higher proportion of patients were prescribed renin-angiotensin system inhibitors at discharge (data not shown). Collectively, the patients with higher BMI were associated with lower severity of HF and fewer comorbidities and were well treated with medication. Such features might be related to the greater advantage of achieving the THR.

### 4.4. Limitations

The present study has some limitations. First, this was a retrospective study based on small observational registry data. Furthermore, the decision on the prescription of HR-lowering agents, including β-blockers, amiodarone, and digoxin, and their doses were made by the attending physicians. Second, since this study involved only Japanese subjects, our findings might not be applicable in other countries. Third, ivabradine was not yet commercially available in Japan during the study period; we could not determine the influence of ivabradine administration in the HFrEF population. Fourth, the validity of THR from a physiological aspect of the mechanism of THR discriminating the outcome of the study participants cannot be explored from this study’s findings. Fifth, as mentioned above, discharge HR was utilized in the present study. Still, it is possible that medications including HR-lowering agents were not yet optimized during the index hospitalization since the WET-HF registry enrolled patients hospitalized for ADHF. Sixth, there are no data on the trajectory of HR or LV geometry (e.g., reverse remodeling) after discharge. Fifth, the atrioventricular delay can affect MV inflow [19], but data on the AV block or PQ interval on the electrocardiogram were not obtained. Lastly, the optimal HR for patients with HF with preserved EF (HFpEF) has also been of interest. However, the formula of THR was based on the results obtained from patients with HFrEF; [10] it is uncertain whether this formula can be applied to HFpEF. According to these limitations, further studies are needed to validate our findings in a large multicenter cohort.

## 5. Conclusions

THR was of use for discriminating outcomes for patients with HFrEF who were hospitalized due to ADHF. Further investigation is warranted to determine whether the strategy aimed at achieving THR benefits patients with HFrEF.

## Figures and Tables

**Table 1:** Baseline characteristics of the study population.

Variable	Overall (N = 647)	L Group (N = 328)	H Group (N = 319)	*p*-Value
Demographics				
Age, years	72 (59, 80)	71 (58, 80)	72 (60, 81)	0.21
Sex (Male)	452 (70%)	242 (74%)	210 (66%)	0.028
BMI, kg/m^2^	22.9 (20.3, 25.7)	23.2 (20.8, 25.9)	22.1 (19.9, 25.1)	0.017
Etiology DCM/ICM/VHD	191/272/72 (30%/42%/11%)	99/142/26 (30%/43%/8%)	92/130/46 (29%/41%/14%)	0.066
Medical history				
History of ADHF hospitalization	200 (31%)	97 (30%)	103 (32%)	0.47
HT	422 (65%)	209 (64%)	213 (67%)	0.42
DLp	289 (45%)	142 (44%)	147 (47%)	0.49
DM	266 (41%)	134 (41%)	132 (41%)	0.89
Smoking	302 (48%)	155 (49%)	147 (47%)	0.65
HD	21 (3%)	8 (2%)	13 (4%)	0.24
COPD	27 (4%)	11 (3%)	16 (5%)	0.30
HOT	23 (4%)	13 (4%)	10 (3%)	0.57
Stroke/TIA	78 (12%)	28 (9%)	50 (16%)	0.005
Pacemaker	33 (5%)	13 (4%)	20 (6%)	0.18
ICD	37 (6%)	26 (8%)	11 (3%)	0.013
CRT	18 (3%)	12 (4%)	6 (2%)	0.17
Clinical profiles at admission				
NYHA (II–III/IV [%])	342/297 (54%/46%)	168/155 (52%/48%)	174/142 (55%/45%)	0.44
SBP	136 (114, 160)	134 (112, 160)	140 (116, 159)	0.71
DBP	81 (68, 99)	81 (66, 98)	82 (70, 100)	0.77
HR	95 (78, 110)	90 (72, 108)	99 (80, 110)	0.006
Labs at admission				
BNP	937 (482, 1592)	951 (468, 1628)	913 (494, 1592)	0.94
NT-proBNP	5727 (3332, 12,357)	5544 (3065, 11,379)	6580 (3732, 14,561)	0.29
Alb	3.7 (3.3, 4.0)	3.7 (3.4, 4.1)	3.6 (3.3, 3.9)	0.026
Hb	12.6 (11.1, 14.4)	12.9 (11.2, 14.6)	12.4 (10.9, 14.0)	0.030
BUN	21.4 (16.3, 30.7)	22.2 (16.6, 31.2)	20.9 (16.1, 30.0)	0.91
Cr	1.1 (0.8, 1.4)	1.1 (0.9, 1.4)	1.0 (0.8, 1.5)	0.41
eGFR	51.2 (35.1, 65.1)	50.8 (36.7, 64.1)	52.7 (32.1, 66.8)	0.76
UA	6.8 (5.6, 8.4)	7.1 (5.7, 8.5)	6.7 (5.5, 8.0)	0.09
Na	140 (137, 142)	140 (137, 142)	140 (137, 142)	0.84
TB	0.8 (0.6, 1.3)	0.9 (0.6, 1.4)	0.8 (0.6, 1.1)	0.007
Echocardiography				
LVDd	59 (53, 65)	59 (55, 65)	59 (52, 65)	0.29
LVDs	51 (45, 57)	51 (46, 58)	51 (44, 57)	0.48
LVEF	30 (23, 35)	29 (23, 35)	30 (23, 35)	0.28
LAD	42 (37, 47)	44 (38, 48)	42 (37, 46)	0.004
E/e’	17.2 (10.8–24.7)	18.0 (11.0–26.0)	16.7 (10.5–23.6)	0.12
TRPG	28 (21–36)	29 (22–37)	27 (19–35)	0.014
Dct	152 (121, 190)	140 (114, 170)	176 (137, 218)	<0.001
THR	73 (68, 77)	75 (71, 78)	70 (65, 75)	<0.001

BMI, body mass index; DCM, dilated cardiomyopathy; ICM, ischemic cardiomyopathy; VHD, valvular heart disease; ADHF, acute decompensated heart failure; HTN, high blood pressure; DLp, dyslipidemia; DM, diabetes mellitus; HD, hemodialysis; COPD, chronic obstructive pulmonary disease; HOT, home oxygen therapy; TIA, transient ischemic attacks; ICD, implantable cardioverter defibrillator; CRT, cardiac resynchronization therapy; NYHA, New York Heart Association functional class; SBP, systolic blood pressure; DBP diastolic blood pressure; HR, heart rate; BNP, B-type natriuretic peptide level; NT-pro BNP, N-terminal pro B-type natriuretic peptide level; Alb, serum albumin level; Hb, hemoglobin level; BUN, blood urea nitrogen level; Cr, serum creatinine level; eGFR, estimated glomerular filtration rate; UA, uric acid level; Na, serum sodium level; TB, total bilirubin level; LVDd, left ventricular end-diastolic diameter; LVD, left ventricular end-systolic diameter; LAD, left atrial diameter; TRPG, tricuspid regurgitation pressure gradient; Dct, deceleration time; THR, target HR.

**Table 2:** Characteristics of the study population at discharge.

Variable	Overall (N = 647)	L Group (N = 328)	H Group (N = 319)	*p* Value
Clinical profiles at discharge				
SBP	108 (96, 120)	106 (94, 119)	107 (96, 120)	0.18
HR	72 (64, 80)	66 (60, 71)	80 (74, 86)	<0.001
Length of hospital stay	16 (11–25)	16 (11–25)	17 (11–26)	0.99
Medication at discharge				
β-blocker	566 (87.5%)	295 (89.9%)	271 (85.0%)	0.055
β-blocker dose (mg carvedilol)	2.5 (1.25, 6.25)	3.75 (1.25, 7.5)	2.5 (1.25, 5)	0.085
β-blocker dose/kg BW	0.057 (0.025, 0.117)	0.059 (0.026, 0.122)	0.055 (0.024, 0.107)	0.19
RAS inhibitor	455 (70%)	232 (71%)	223 (70%)	0.82
MRA	297 (46%)	145 (44%)	152 (48%)	0.40
Amiodarone	82 (13%)	52 (16%)	30 (9%)	0.013
Digoxin	9 (1.4%)	7 (2%)	2 (0.6%)	0.09
Loop diuretics	500 (77%)	258 (79%)	242 (76%)	0.36
Loop diuretics (dose, mg)	20 (20–40)	20 (20–40)	20 (20–40)	0.69
Tolvaptan	34 (6.0%)	13 (4.4%)	22 (7.4%)	0.13

SBP, systolic blood pressure; HR, heart rate; RAS, renin-angiotensin system; MRA, mineralocorticoid antagonist.

## Data Availability

The data underlying this article cannot be shared publicly to maintain the privacy of individuals that participated in the study. The data will be shared on reasonable request to the corresponding author.

## Supplementary Materials

The following supporting information can be downloaded at https://www.mdpi.com/article/10.3390/jpm12010050/s1, Figure S1: Distribution of THR in the (A) overall population, (B) L group, and (C) H group, THR, target HR. Figure S2: Hazard ratios of the L/H group for the primary endpoint adjusted for the covariates in the subgroups. BMI, body mass index; ICM, ischemic cardiomyopathy; NICM, non-ischemic cardiomyopathy; DM, diabetes mellitus; NYHA, New York Heart Association functional class; SBP, systolic blood pressure; eGFR, estimated glomerular filtration rate; LVEF, left ventricular ejection fraction; THR, target HR.

## References

[1] Shiraishi Y., Kohsaka S., Sato N., Takano T., Kitai T., Yoshikawa T., Matsue Y. (2018). 9-Year Trend in the Management of Acute Heart Failure in Japan: A Report from the National Consortium of Acute Heart Failure Registries. J. Am. Heart Assoc..

[2] Takada T., Sakata Y., Miyata S., Takahashi J., Nochioka K., Miura M., Tadaki S., Shimokawa H. (2014). Impact of elevated heart rate on clinical outcomes in patients with heart failure with reduced and preserved ejection fraction: A report from the CHART-2 Study. Eur. J. Heart Fail..

[3] McAlister F.A., Wiebe N., Ezekowitz J.A., Leung A.A., Armstrong P.W. (2009). Meta-analysis: Beta-blocker dose, heart rate reduction, and death in patients with heart failure. Ann. Intern. Med..

[4] Kotecha D., Flather M.D., Altman D.G., Holmes J., Rosano G., Wikstrand J., Packer M., Coats A.J.S., Manzano L., Böhm M. (2017). Heart Rate and Rhythm and the Benefit of Beta-Blockers in Patients with Heart Failure. J. Am. Coll. Cardiol..

[5] Nagatomo Y., Yoshikawa T., Okamoto H., Kitabatake A., Hori M. (2020). Differential Response to Heart Rate Reduction by Carvedilol in Heart Failure and Reduced Ejection Fraction between Sinus Rhythm and Atrial Fibrillation—Insight from J-CHF Study. Circ. Rep..

[6] Swedberg K., Komajda M., Böhm M., Borer J.S., Ford I., Dubost-Brama A., Lerebours G., Tavazzi L. (2010). Ivabradine and outcomes in chronic heart failure (SHIFT): A randomised placebo-controlled study. Lancet.

[7] Böhm M., Swedberg K., Komajda M., Borer J.S., Ford I., Dubost-Brama A., Lerebours G., Tavazzi L. (2010). Heart rate as a risk factor in chronic heart failure (SHIFT): The association between heart rate and outcomes in a randomised placebo-controlled trial. Lancet.

[8] Chung C.S., Kovács S.J. (2006). Consequences of increasing heart rate on deceleration time, the velocity-time integral, and E/A. Am. J. Cardiol..

[9] Appleton C.P. (1991). Influence of incremental changes in heart rate on mitral flow velocity: Assessment in lightly sedated, conscious dogs. J. Am. Coll. Cardiol..

[10] Izumida T., Imamura T., Nakamura M., Fukuda N., Kinugawa K. (2020). How to consider target heart rate in patients with systolic heart failure. ESC Heart Fail..

[11] McKee P.A., Castelli W.P., McNamara P.M., Kannel W.B. (1971). The natural history of congestive heart failure: The Framingham study. N. Engl. J. Med..

[12] Simon L., Ghaleh B., Puybasset L., Giudicelli J.F., Berdeaux A. (1995). Coronary and hemodynamic effects of S 16257, a new bradycardic agent, in resting and exercising conscious dogs. J. Pharmacol. Exp. Ther..

[13] Sato M., Hoka S., Arimura H., Ono K., Yoshitake J. (1991). Effects of augmenting cardiac contractility, preload, and heart rate on cardiac output during enflurane anesthesia. Anesth. Analg..

[14] Chung C.S., Karamanoglu M., Kovács S.J. (2004). Duration of diastole and its phases as a function of heart rate during supine bicycle exercise. Am. J. Physiol. Heart Circ. Physiol..

[15] Kusunose K., Arase M., Zheng R., Hirata Y., Nishio S., Ise T., Yamaguchi K., Fukuda D., Yagi S., Yamada H. (2021). Clinical Utility of Overlap Time for Incomplete Relaxation to Predict Cardiac Events in Heart Failure: Incomplete relaxation in heart failure. J. Card. Fail..

[16] Izumida T., Imamura T., Ueno Y., Tanaka S., Kataoka N., Nakamura M., Kinugawa K. (2021). Impact of optimal heart rate on left ventricular reverse remodeling and functional improvement in patients with systolic heart failure. Heart Vessel..

[17] St Goar F.G., Masuyama T., Alderman E.L., Popp R.L. (1991). Left ventricular diastolic dysfunction in end-stage dilated cardiomyopathy: Simultaneous Doppler echocardiography and hemodynamic evaluation. J. Am. Soc. Echocardiogr. Off. Publ. Am. Soc. Echocardiogr..

[18] DeSanctis R.W. (1971). Diagnostic and therapeutic uses of atrial pacing. Circulation.

[19] Inoue N., Ishikawa T., Sumita S., Nakagawa T., Kobayashi T., Matsushita K., Matsumoto K., Ohkusu Y., Taima M., Kosuge M. (2005). Long-term follow-up of atrioventricular delay optimization in patients with biventricular pacing. Circ. J..

