# Supplementary material for: Personalized Target Heart Rate for Patients with Heart Failure and Reduced Ejection Fraction
Source: J Pers Med. 2022 Jan 5;12(1):50. doi: 10.3390/jpm12010050 (PMC8777886; doi:10.3390/jpm12010050)
Supplement: Supplementary file 1 [file jpm-12-00050-s001.zip › JPM Supplemental Figures.pdf]

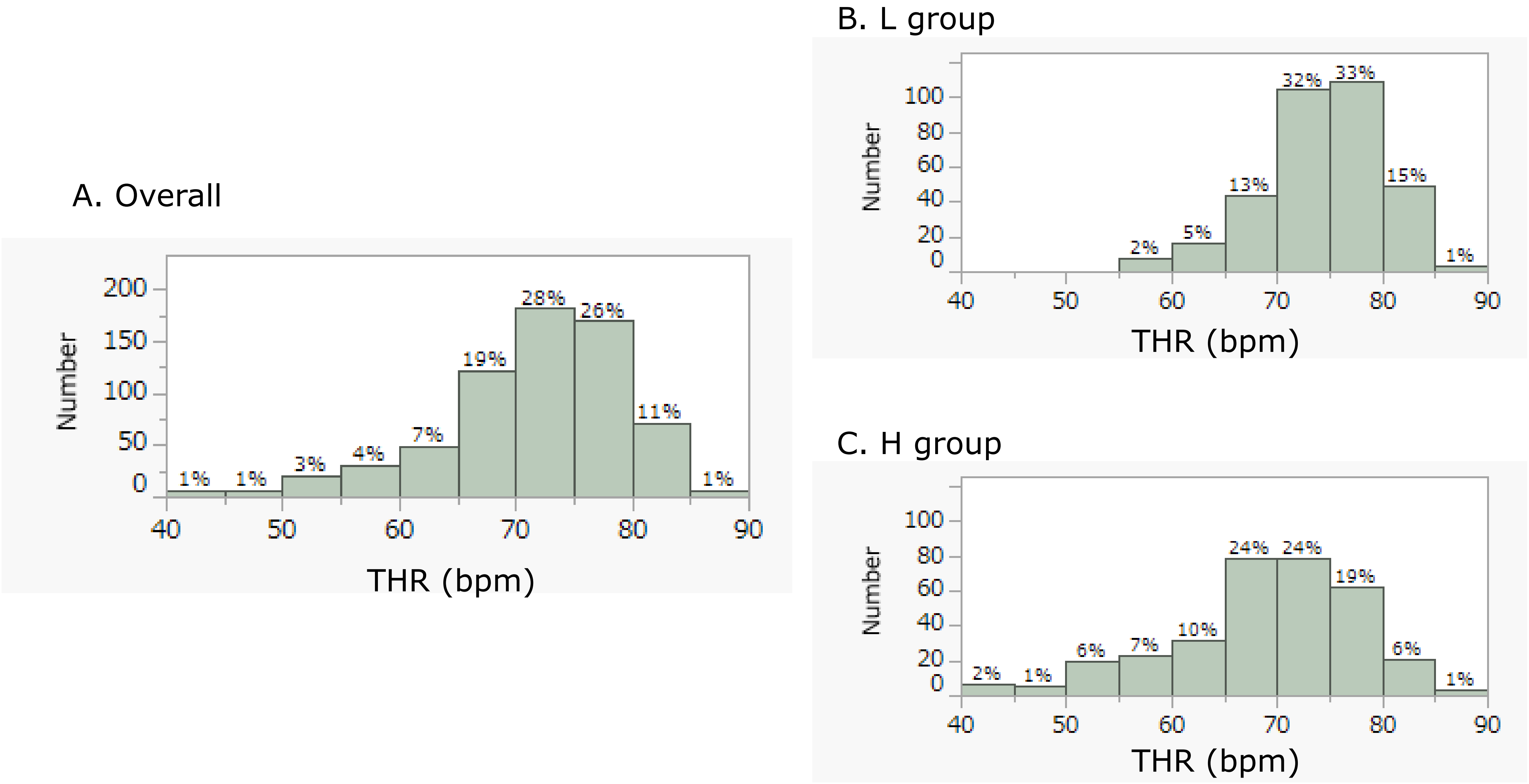

Supple. Figure S1 Distribution of THR in the L group and the H group

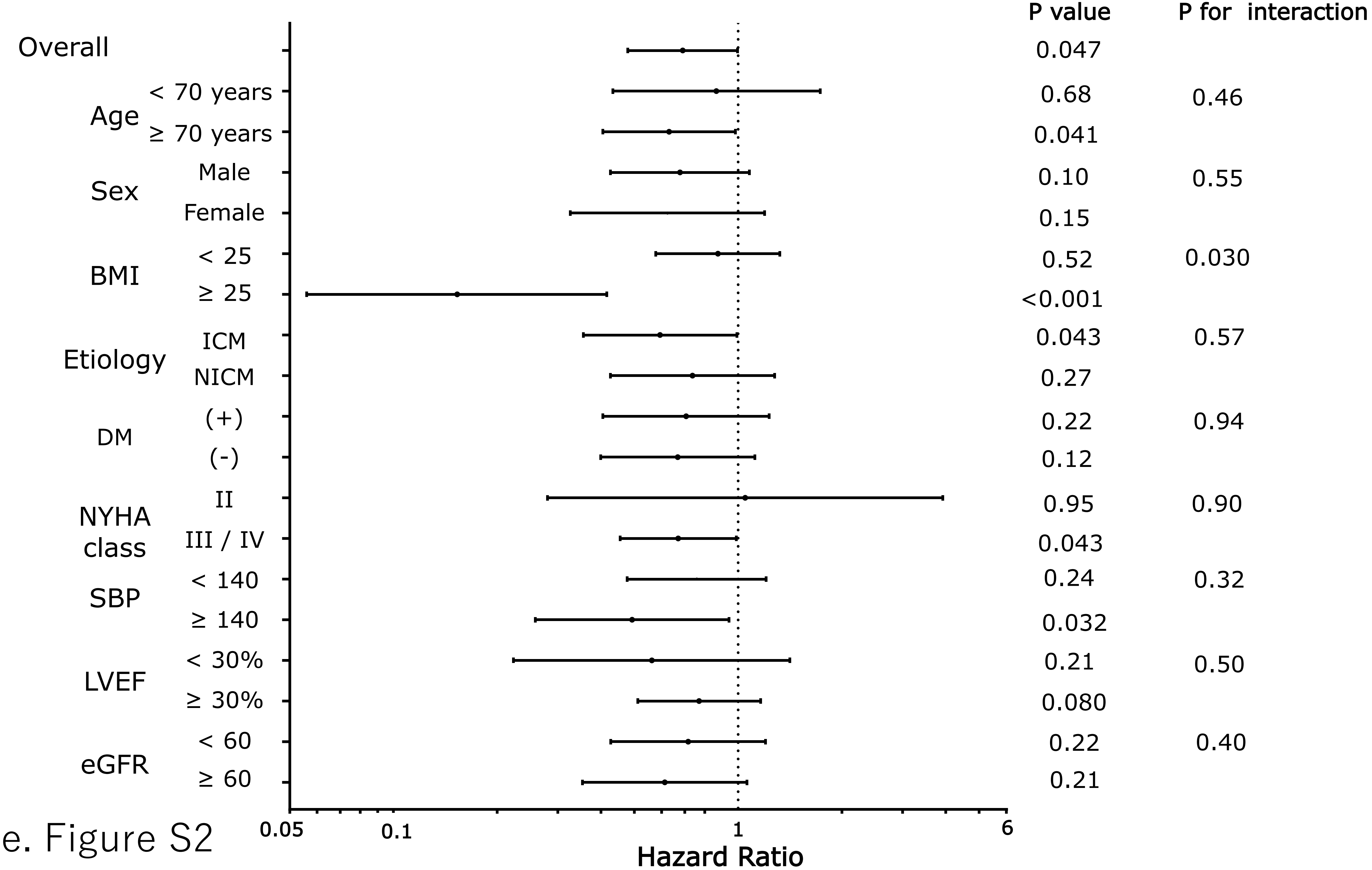

Supple. Figure S2
